# Heel reconstruction with ALT free flap in a 4-year-old patient after a severe lawnmower injury. A case report

**DOI:** 10.1080/23320885.2022.2157280

**Published:** 2022-12-28

**Authors:** Emilio Trignano, Pietro Luciano Serra, Federica Grieco, Manuela Rodio, Silvia Rampazzo, Nicola Pili, Claudia Trignano, Corrado Rubino

**Affiliations:** a Department of Medical, Surgical and Experimental Sciences, Plastic Surgery Unit, Sassari University Hospital Trust, University of Sassari, Sassari, Italy; b Department of Biomedical Sciences, University of Sassari, Sassari, Italy

**Keywords:** Lawnmower, trauma, flap, reconstruction, pediatric, microsurgery

## Abstract

Lawnmowers represent a danger in pediatric population. Frequently, traumas involve limbs. Among the different reconstructive techniques, a free flap is often needed.

We discuss the first case of heel reconstruction with an anterolateral thigh flap in a 4-years-old patient after a lawnmower’s trauma.

## Introduction

Although stricter safety specifications and product designs have been changing over the past few years, lawnmowers continue to be dangerous and a source of serious morbidity, especially in pediatric population. According to Bachier et al. lawnmower’s incidence in patients younger than 20 years old is estimated to be 9400 per year [1] and lower extremities are the second body part most involved in these injuries.

Foot and, in particular, heel reconstruction represents a challenge for plastic surgeons, not only to reinstate an aesthetic appearance but also a proper function [2,3].

Different reconstructive techniques have been described: negative pressure wound therapy (NPWT) or acellular dermal matrixes (ADMs) with skin grafts, local flaps or free flaps [3,4].

Nowadays free tissue transfer is recognized as a reliable reconstructive technique not only in adults but also in the pediatric population [5].

In our paper, we report the first case in literature of heel reconstruction after a severe lawn-mower injury using a free anterolateral thigh (ALT) flap in a 4-year-old patient.

## Case presentation

**Figure 1.**
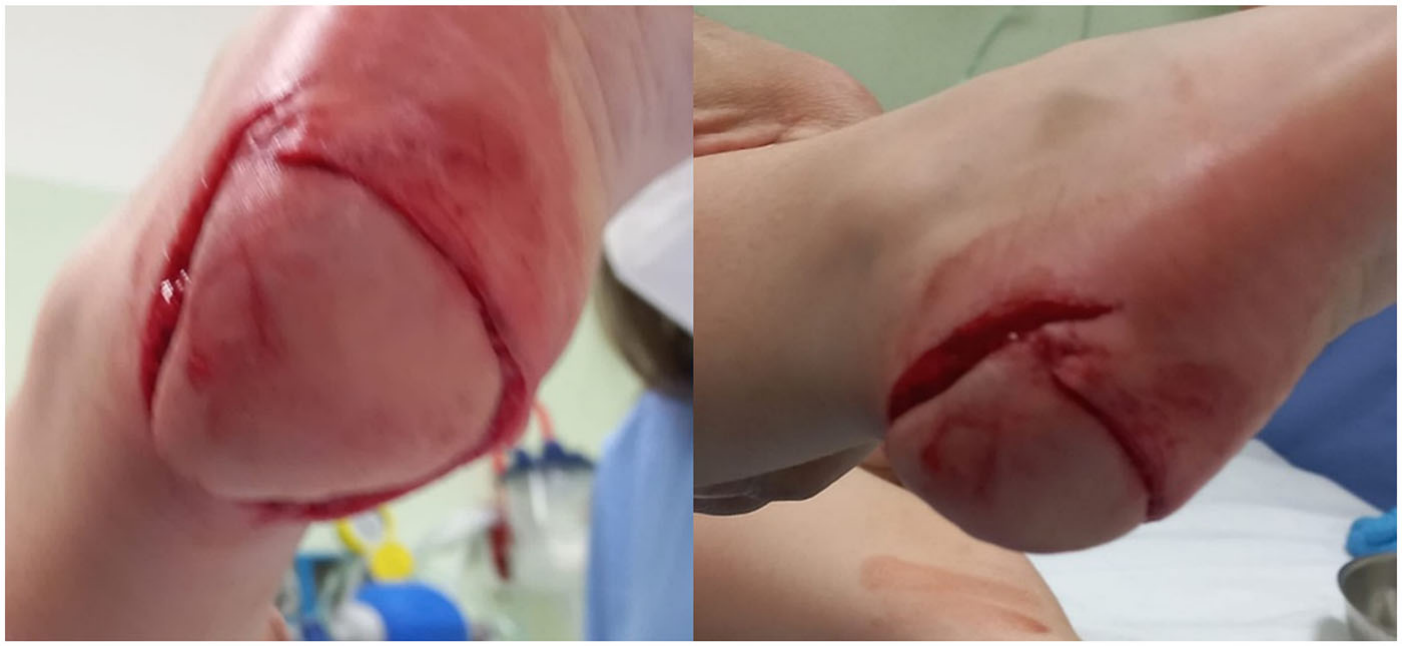
Preoperative photos.

A 4-year-old patient suffered from a domestic trauma caused by a lawnmower resulting in a sub-amputation of the right foot heel plantar skin and subcutaneous tissue (Figure 1). After trauma, the child was brought to the closest emergency department where an initial assessment was performed: GCS was evaluated as 15 and no additional injury or comorbidities were reported.

No signs of bone, tendon, or vascular injury nor any deficit of foot movements of the right foot were detected during the orthopedic surgeon evaluation, thus no X-ray was done. The patient was therefore referred to our Plastic Surgery Unit.

At the clinical examination, a 4 × 5 cm (area 20 cm^2^) plantar heel skin flap, affecting more than half of the weight-bearing area, was attached to the rest of the foot by a thin postero-lateral cutaneous pedicle.

A conservative approach with a suture (Dafilon 4/0) of the plantar skin was attempted under general anesthesia.

After 5 days, only about 15% of the replanted skin survived. Hence, another surgical procedure under general anesthesia was planned.

**Figure 2.**
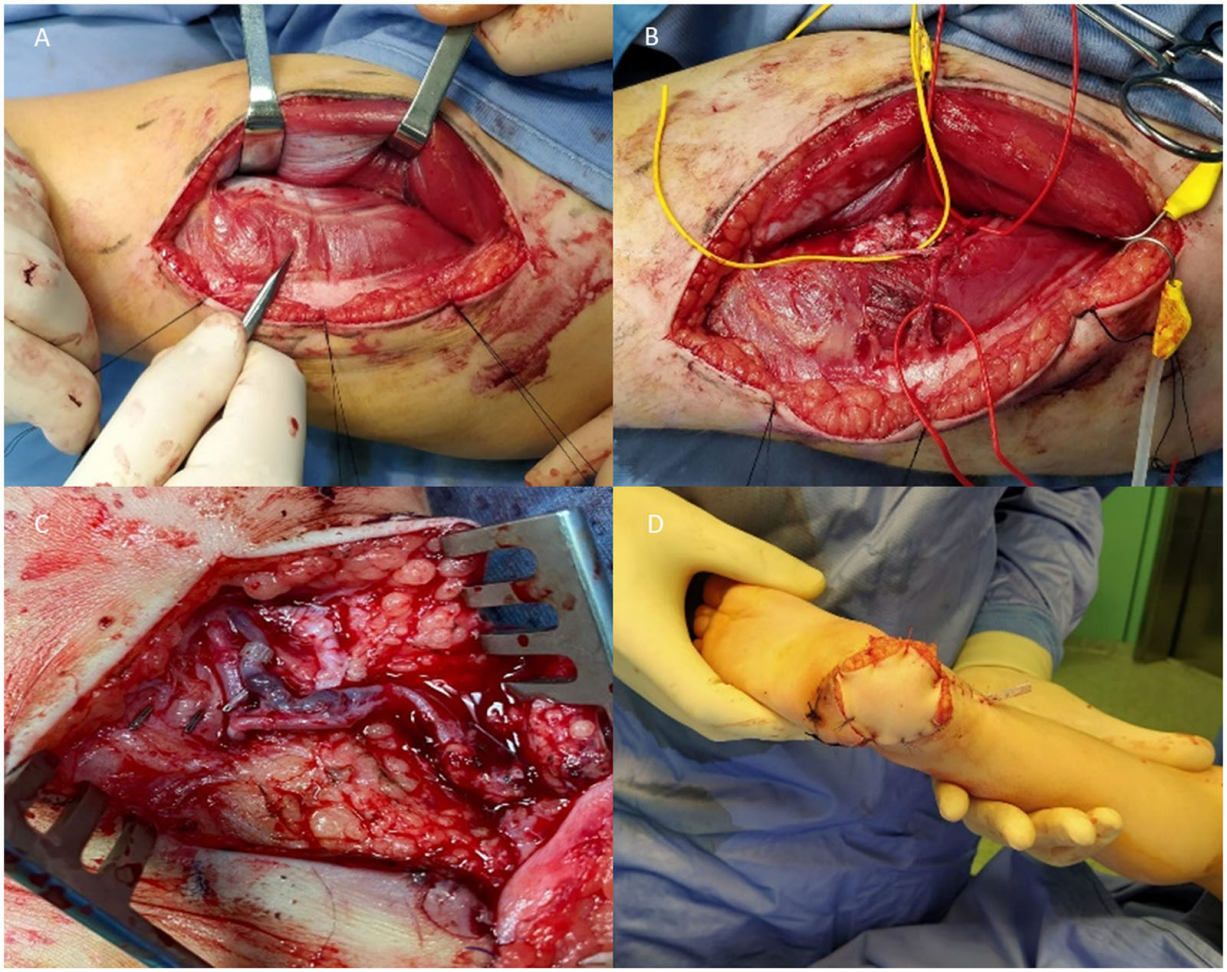
Intraoperative photos. (A) The perforator of the descending branch of lateral circumflex femoral artery is shown between rectus femoris and vastus lateralis muscle. (B) The perforator is isolate. (C) Anastomosis. (D) Suture of the flap.

First, an accurate debridement was performed so that all the necrotic tissue was removed, leaving a 4.5 × 5.5 (25 cm^2^) soft tissue defect that could not be closed primarily or with local flaps. Posterior tibial artery and veins were then identified and prepared as recipient vessels. A 5 × 6 cm (30 cm^2^) ALT flap was elevated based on one septal perforator of the descendent branch of the lateral circumflex femoral artery. An arterial side-to-end anastomosis and a venous end-to-end anastomosis (1.5 mm coupler) were performed. We applied a ‘Penrose’ drainage and used a single-stitch suture (Figure 2). The donor site was closed primarily.

Total surgery time was 210 min and flap ischemia 40 min. A plaster cast was used for 10 days in order to avoid any movement of the foot. Post-operative period was uneventful and hospitalization lasted 6 days. No rehabilitation program was planned due to his age.

Follow-up visits for medications were fixed at 9, 12, 15, 18 and 21 days after surgery.

At 2 weeks, the flap survived successfully, and stitches were removed; the donor site was healed, with a 15 cm linear vertical scar on the left tight, normochromic and normotrophic, with a regular evolutionary course and without signs of infection or dehiscence. The presence of scars from the flap in weight-bearing area didn’t impact on functionality: in fact, after just 6 weeks the patient started walking again and was able to wear shoes. No deficit in moving ankle and dorsiflexion of the foot was detected.

**Figure 3.**
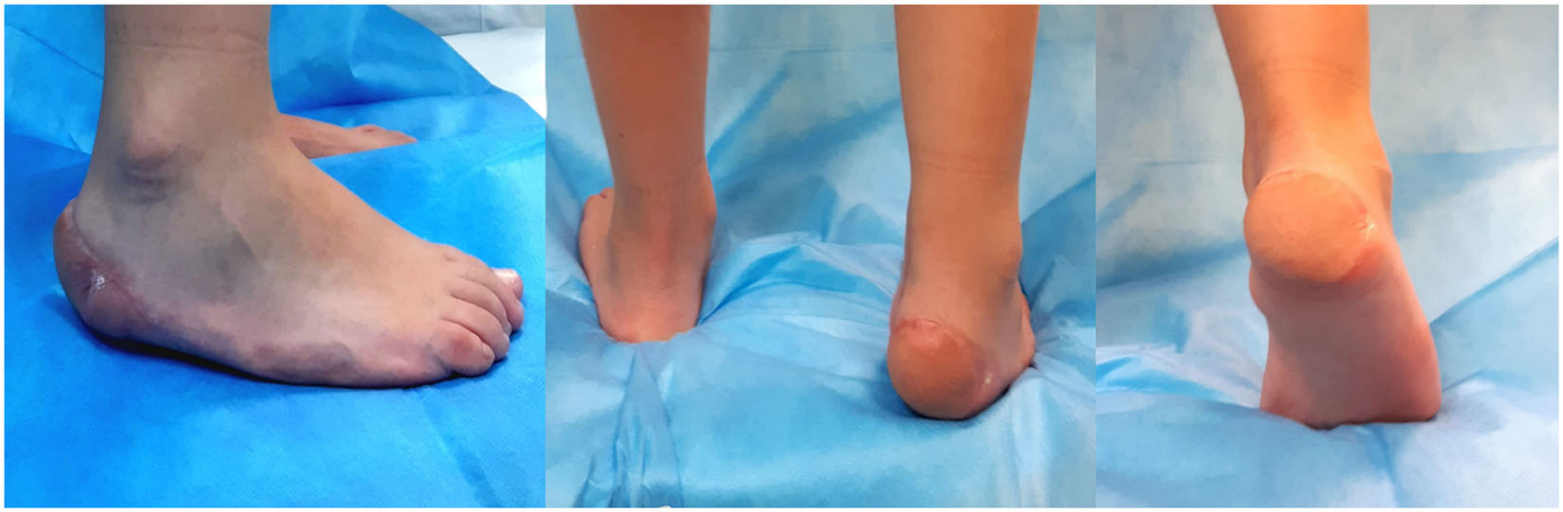
Postoperative photos, 18 months.

After 18 months of follow-up, the aesthetic outcome was good, and no ulceration occurred in the flap (Figure 3). Neither in the long period, the scars from the flap in weight-bearing area didn’t create any functional deficit or delay in recovery times.

The functional outcome in the long period was evaluated at 18 months with the Lower Extremity Functional Scale (LEFS) giving excellent results, with a total score of 78/80 points, corresponding to 97.5% of maximal function [6].

Postoperative evaluations also considered the time interval to return to daily living activities (bearing weight on the reconstructed surface for at least 5 h per day), that was about 2 months, and empiric tests for the sensitive function, such as sensation to light touch (+), pinprick test (+), Semmes- Weinstein monofilament test for sensory evaluation [4–7].

## Discussion

Heel reconstruction is a field of interest in plastic surgery because of the peculiarity of the anatomical region.

The undersurface of the foot represents a very important area of body weight transmission, with the heel and the underlying calcaneus conveying 80% of it and the distal sole with the metatarsal heads conveying the other 20% [8]

Due to its important role, the weight-bearing surface of the foot has specific anatomical features: a thick epidermis, a minimal dermis, a soft subcutaneous tissue and dense septae adhering skin to the underlying fascia. This shock-absorbing pad maintains a constant pressure and protects calcaneus from shear forces [9].

Many soft-tissue reconstructive techniques of the calcaneal region have been described in order to restore these properties: NPWT or ADM with skin graft [10]; local flaps [11,12]; local pedicled flaps [12] and free flaps [13].

NPWT as “a bridge therapy”, keeping the patient immobilized for at least 3–4 weeks, would not have been possible in such a young patient.

Hence, we considered to cover the defect directly with a flap. The instep flap based on the deep branch of the medial plantar artery would have been a valid option for heel reconstruction, as much as a medial plantar perforator flap based on the perforators of the superficial branch of the medial plantar artery [3].

Both these flaps would have offered a minimal donor-site morbidity and, mostly, they would have best applied the basic plastic surgery principle to replace “like with like”.

However, choosing an instep flap or a medial plantar perforator flap, in both cases a skin graft would have been necessary to cover the donor site, and it would have caused skin retraction compromising foot’s growth, with additional scars for the child on the donor site of the skin. Secondly the heel plantar skin and subcutaneous tissue area involved in the defect was too wide to be covered and repaired by this kind of flaps. Moreover, because of the patient’s age, a further problem would be represented by the reduced caliber of the vessels, so we would have gone through a tricky perforator’s dissection, especially in performing a medial plantar perforator flap.

We oriented then towards a free tissue transfer from a different anatomical area.

Among muscle flaps, we decided not to perform a gracilis flap because of the short vessels; furthermore, our defect was too big to be covered. Nevertheless, a latissimus dorsi flap would have been oversized and it would have significantly impaired patient’s life. Regarding fasciocutaneous flaps, TDAP [14] would have represented a valid solution in adults but anatomical variations and perforators’ caliber would have made the surgery too risky in a pediatric patient. Moreover, radial forearm flap would have been too thin, and it would have required additional skin graft to cover the donor site.

Under these circumstances, we decided to perform an ALT free flap, using an arterial side-to-end anastomosis in order to preserve the distal foot circulation.

We avoided a sensate flap since recent studies reported that neurosensorial flaps were not superior to the non-innervate flaps in a weight-bearing area [6,15].

In fact, despite has been demonstrated that the use of sensate flaps offers the advantage of a faster return to daily living activities for the patient (often without the necessity of wearing a protective shoe) however, in the long run, also patients with non-sensate flaps reconstruction reported no difficulties with their daily routine. These satisfactory results also for non-sensate flaps can be explained by secondary neurotization and proprioception of the injured areas, thus progressively returning to a partial protective sensation, and by having intact pressure zones of the plantar region on which patient can rely on [7].

Furthermore, in our case we had a relatively small size flap, which can benefit from a possibly faster reinnervation from the periphery, and above all we considered in our choice the note greater capacity of nervous regeneration in pediatric age. Moreover, we avoided a sensate flap to prevent technical difficulties due to perform nervous coaptation between very small structures in the child.

Since the trauma resulted in calcaneal sub-amputation involving heel plantar skin and subcutaneous tissue without any damage on the underlying bone, a composite flap which also provided the bone component was not required in this circumstance [16].

Finally, for the flap closure, we opted for a single-stitches suture in order to avoid a compression of the flap and consequent onset of venous hypertension [17].

As a matter of fact, microsurgical procedures in pediatric patients have developed more slowly than in adults [16]. Surgeons were initially sceptic to use free flaps in children because the minute vessel diameters, perceived vessel spasticity, and limited tissue availability. Despite nowadays microsurgery is performed also in pediatric population, relatively little has been published in literature regarding this topic [5]. Our case represents the youngest patient who underwent heel reconstruction with an ALT free flap after a lawnmower’s domestic accident [18,19].
